# Early prediction of hypothermia in pediatric intensive care units using machine learning

**DOI:** 10.3389/fphys.2022.921884

**Published:** 2022-09-02

**Authors:** Pradeep Singh, Aditya Nagori, Rakesh Lodha, Tavpritesh Sethi

**Affiliations:** ^1^ Indraprastha Institute of Information Technology, Delhi, India; ^2^ CSIR-Institute of Genomics and Integrative Biology, New Delhi, India; ^3^ Academy of Scientific and Innovative Research (AcSIR), Ghaziabad, India; ^4^ All India Institute of Medical Sciences, Department of Pediatrics, New Delhi, India

**Keywords:** hypothermia, artificial intelligence, prospective validation, pediatric intensive care unit, time-series

## Abstract

Hypothermia is a life-threatening condition where the temperature of the body drops below 35°C and is a key source of concern in Intensive Care Units (ICUs). Early identification can help to nudge clinical management to initiate early interventions. Despite its importance, very few studies have focused on the early prediction of hypothermia. In this study, we aim to monitor and predict Hypothermia (30 min-4 h) ahead of its onset using machine learning (ML) models developed on physiological vitals and to prospectively validate the best performing model in the pediatric ICU. We developed and evaluated ML algorithms for the early prediction of hypothermia in a pediatric ICU. Sepsis advanced forecasting engine ICU Database (SafeICU) data resource is an in-house ICU source of data built in the Pediatric ICU at the All-India Institute of Medical Science (AIIMS), New Delhi. Each time-stamp at 1-min resolution was labeled for the presence of hypothermia to construct a retrospective cohort of pediatric patients in the SafeICU data resource. The training set consisted of windows of the length of 4.2 h with a lead time of 30 min-4 h from the onset of hypothermia. A set of 3,835 hand-engineered time-series features were calculated to capture physiological features from the time series. Features selection using the Boruta algorithm was performed to select the most important predictors of hypothermia. A battery of models such as gradient boosting machine, random forest, AdaBoost, and support vector machine (SVM) was evaluated utilizing five-fold test sets. The best-performing model was prospectively validated. A total of 148 patients with 193 ICU stays were eligible for the model development cohort. Of 3,939 features, 726 were statistically significant in the Boruta analysis for the prediction of Hypothermia. The gradient boosting model performed best with an Area Under the Receiver Operating Characteristic curve (AUROC) of 85% (SD = 1.6) and a precision of 59.2% (SD = 8.8) for a 30-min lead time before the onset of Hypothermia onset. As expected, the model showed a decline in model performance at higher lead times, such as AUROC of 77.2% (SD = 2.3) and precision of 41.34% (SD = 4.8) for 4 h ahead of Hypothermia onset. Our GBM(gradient boosting machine) model produced equal and superior results for the prospective validation, where an AUROC of 79.8% and a precision of 53% for a 30-min lead time before the onset of Hypothermia whereas an AUROC of 69.6% and a precision of 38.52% for a (30 min-4 h) lead time prospective validation of Hypothermia. Therefore, this work establishes a pipeline termed *ThermoGnose* for predicting hypothermia, a major complication in pediatric ICUs.

## 1 Introduction

Hypothermia is a major complication associated with sepsis in the ICUs and is especially in children (Rumbus et al., 2017). Hypothermia is a situation when the human body temperature is below 35°C (95°F) (Fears, 2011). It arises when the human body loses more heat compared to what it is producing, thus leading to a drop in the core body temperature. There may be some warning signs such as Shivering, Confusion, and Slurred speech, whereas, in the worst scenario, this may eventually lead to organ failure and may also cause death. In resource-limited situations, hypothermia is increasingly identified as a major cause of newborn illness and mortality (Ibrahim et al., 2021). Also, children are particularly vulnerable to hypothermia than adults because children have a larger body surface area in proportion to their bodily weight, allowing them to lose heat more quickly (Singer, 2021).

Pathophysiology of Hypothermia involves cold diuresis which can result in loss of fluid culminating in the risk of hypovolemia (Brown et al., 2012). The treatment for this circulatory malfunction involves medication with vasopressors. A repeated and prolonged usage of these medications may result in worsened outcomes (Martin et al., 2015; Vincent et al., 2018). A regulated rewarming is recommended in hypothermia; however, a quick rewarming can result in a shift in electrolyte balance of the body resulting in arrhythmias and cardiac arrest (Dietrichs et al., 2020), therefore a decision support system for an early assessment is required for regulating the rewarming cycles.

In critically ill patients, the temperature is not only an important clinical indication of illness severity but is also an independent predictor of morbidity and mortality. Hypothermia may be a significant and modifiable factor linked to a higher risk of death in critically ill individuals. Close monitoring and regulation of body temperature to minimize extremes are especially vital in severely ill patients (Faulds and Meekings, 2013). Also, preventive measures must be taken to avoid the grueling consequences of hypothermia (Paal et al., 2018). A poor prognosis can increase the risk of Hypothermia occurrence and associated complications. Thus, to avoid delayed identification, Hypothermia monitoring needs to be advanced with the use of artificial intelligence (AI). AI is already transforming medicine, where algorithms have been surpassing the clinical accuracy of disease prediction in ICU (Bohr and Memarzadeh, 2020). However, none of the studies, to our knowledge, have developed Hypothermia prediction models despite the higher association of mortality with hypothermia (Fatteh et al., 2021). Early detection using predictive modeling of hypothermia can save lives.

Artificial intelligence algorithms such as machine-learning can automatically rebuild associations between variables and response values from big data and enhance the interpretation of conventional techniques, such as support vector machines, random forest algorithms, and regression techniques in pinpointing crucial predictors (Jiang et al., 2017).

The abundance of continuous monitor data is complemented by applicable data from the EHR (electronic health record) or the increasingly widespread wireless wearable devices that assess physiologic signals (Rush et al., 2019). The analytic tools of machine learning can both help the workflow of reading vital signs and provide insights into data patterns and complexities beyond the perceptual capacity of the average human clinical observer (Chen and Asch, 2017; Obermeyer and Lee, 2017). In data-rich situations, machine-learning (ML) algorithms excel at analysing complicated signals (Krumholz, 2014; Tomašev et al., 2019).

Disease categorization and prediction models can be improved using machine learning techniques. These methods might be beneficial in the clinic for automatically identifying individuals with extremely morbid illnesses who could benefit from intensive risk factor treatment (Ross et al., 2016). In earlier research, machine learning and statistical modeling techniques were used to address the issues related to sepsis detection and care management (Henry et al., 2015). Several researchers employed machine learning algorithms to identify those who were most likely to die from sepsis (Gultepe et al., 2014; Mayhew et al., 2018; Taylor et al., 2016).

This research developed a real-time hypothermia prediction model using physiological vitals time-series data and ML techniques. We defined the onset of hypothermia as a time-point when core body temperature is <35C. We tested a battery of machine learning models and selected the best one for the prospective validation in our pediatric ICU. Therefore, our study ThermoGnose aimed at building predictive models for hypothermia prediction, which can be used in an ICU setting for real-time decision making.

## 2 Methods

### 2.1 Study data and pre-processing

The ICU data stated in this investigation work were gathered from the Paediatric ICU at AIIMS. The patient’s non-public information was not required amid information warehousing, additionally, there was no modification that drained the patient’s care, consequently, the Ethics committee of the medical institute allowed an assent for this investigation (IEC/NP-211/08.05.2015) (Sethi et al., 2017). No data was discarded during this process as segregation of data was not done. Pediatric ICU consists of eight beds which also includes infant beds. Phenotype deeply, Capture Reliably, enable decisions, and Systemic Approach are the four principles that constitute Sepsis Advanced Forecasting Engine for the ICU’s System. The SafeICU data warehoused between the months of January 2019 to January 2020 contained 193 ICU stays (884 days) from a total of 148 patients. Additionally, the ICU data collected from the month of February 2020 to November 2020 had been used for prospective validation.

#### 2.1.1 Physiological vitals records

Health Level 7 (HL7) Standards-based querying of the multi-parameter Central Monitoring Station (CMS) MindrayTM monitors was extracted using in-house software. Physiological vitals data i.e., Respiratory rate (RR), systolic and diastolic blood pressure (Sys-bp, Dia-bp), Heart-rate (HR), Oxygen saturation (SpO2), and temperature records, were used. T1 and T2 temperature probes were used for the abdomen and foot, respectively. The HL7 format data obtained from the monitoring station was stored on the server. The data were parsed to analyzable tabular forms and pre-processed for analysis and model building. Notification messages generated by Pushbullet™ and RPushbullet have been dispatched to the android phones in case of data loss i.e., on the off chance that there’s any interference in the data streaming.

#### 2.1.2 Treatment-charts

The in-house doctors maintain a proper note of the treatments in the word document. The backup of these files was planned at a few settled hours each day. Parsing of text files was done using the Docx-2-txt python module and then it is converted into the tabular form so as to make it easier for text mining.

### 2.2 Imputation

Pre-processed Safe-ICU stay data were imputed using Kalman Smoothing utilizing the structural time-series (StructTS) model using the imputeTS package in R (Moritz and Bartz-Beielstein, 2017).

### 2.3 Data preprocessing and cohort-generation

#### 2.3.1 Cohort construction

The cohort was constructed using the SafeICU data with a case-crossover design (Navidi, 1998), whereas multiple instances were taken from each subject at 30 min to 4 h ahead of the onset. All patients with continuous vitals monitoring for greater than 4.2 h of their stay in ICU were included for cohort construction. The exclusion criteria included the following. 1) Patients with <4.2 h of ICU stay, 2) patients with non-availability of temperature monitoring within the observation window, or 3) patients with non-availability of age and gender, 4) patients already in hypothermia within the first 4 h of admission based upon temperature recordings 5) patients with missing data in >10% of the observation period.

#### 2.3.2 Data pre-processing and scoring

**FIGURE 1:**
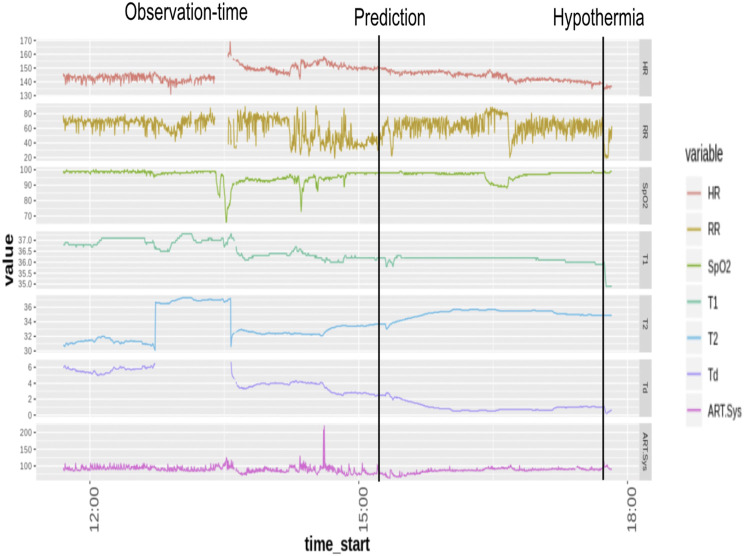
Physiological variables for prediction of Hypothermia in pediatric-ICU.

Temperature (T1), Systolic Arterial Blood-Pressure, Oxygen Saturation, Respiratory Rate, and H eart rate, were utilized as the physiological predictors. Minimal pre-processing of the vitals data was done. Data imputed using the Kalman filter was used for further modeling on the patients eligible for the development cohort. Each timestamp of the vitals time series at a 15-s resolution was evaluated, and binary labels for hypothermia (yes/no) were assigned to 30-min time epochs. The onset time of hypothermia was taken as the start time of an epoch when the median binary label of temperature (T1) value was less than 35 (Musi et al., 2021) (Figure 1). Observational windows of length 256 min were taken 30 min to 4 h ahead of the onset time.

#### 2.3.3 Non-linear times series feature extraction and selection

A set of 3,939 TS features (time-series features) consisting of linear and nonlinear physiological features say Wavelet-transform coefficients, discriminative power, Fourier-transform coefficients, statistics, and other complex features had been extracted utilizing “tsfresh” python-package (Christ et al., 2018). Further, the Boruta feature selection algorithm was utilized to carry out the variable selection. Boruta uses a top-down approach to find important characteristics, comparing the value of original attributes to the importance achievable at random, calculating using their permuted duplicates, and gradually removing unnecessary information. TS features are explainable and also capture physiological domain understanding, as contradicted to black-box features utilizing deep learning. Variable selection was performed using the Boruta algorithm with OOB, which was optimized using grid search for their n-features and n-tree combination, carried out in R.

### 2.4 Model development and evaluation

The development cohort consisted of patients who developed Hypothermia withinside the subsequent 4 h of the index time. The outcome variable is Hypothermia status withinside the subsequent 4 h as described by Hypothermia Binary labels. Five-fold cross-validation sets were built and hyperparameter tuning was performed to attain the best performance on the validation set utilizing grid search. We have named our pipeline as ThermoGnose and have incorporated it in the figure and text (Figure 5). To overcome the problem of class imbalance, we undersampled the majority class for training the models (Bach et al., 2019). The greatest technique for overcoming the problem of class imbalance was to undersample the majority class’s training. The AUROC, Precision, and Recall had been accessed at the test-set. Comparison of model performance were the indicators for different lead times. Normalization of TS features was done by the z-scores computation. Random-Forest (Breiman, 2001), Adaboost (Hastie et al., 2009), Gradient boosting machine (GBM) (Chen and Guestrin, 2016), and the SVM (Noble, 2006) models had been constructed upon TS-features utilizing R libraries (Ihaka and Gentleman, 1996).

### 2.5 Shapley additive exPlanations value analysis for model interpretability

Shapley Additive exPlanations (SHAP) is a method that assigns significant weight to each feature using game-theory principles (Rodríguez-Pérez and Bajorath, 2020). The SHAP values are utilized to describe how features affect the model prediction. Each feature’s SHAP value allocation for all test sets was determined. The top 20 essential features and their relationship with SHAP values were plotted on the test sets. On the test sets, the top 20 essential features and their relationship with SHAP values are plotted.

## 3 Results

### 3.1 Data characteristics

The cohort consisted of 193 ICU stays with 2,723 multiple episodes of hypothermia. Although Pneumonia (24.2%), Shock (23.4%), and Sepsis (19.7%) were the most frequent diagnoses in our cohort, there was a diverse representation of other diagnoses such as Congestive Heart Failure, Tuberculosis, Liver Failure, etc. The complete list of diagnoses and their percentage is listed in the Supplementary Table S1. Further, the relatively high prevalence (28%) of Hypothermia in this cohort reflects the necessity of early preventive measures in pediatric ICU. The temperature, respiratory rate, and oxygen saturation were found to be significantly different 1 h before the onset of Hypothermic and non-hypothermic events. (Table 1). It is evident that the median temperature of the hypothermic subject was 1.17 C lower than the non-hypothermic subject 1 h before its onset, *p*-value = 0.0001, while the respiratory rate was lower in the hypothermia by 1.62 bps, *p*-value 0.0005. Further, we note that the length of stay (LOS) is associated with the length of stay in hypothermia. A 10-min increase in Hypothermia duration can increase the length of stay (LOS) by 68 min (0.0038) in a wide range of patients with comorbidities. This conclusion from our observational design of ThermoGnose will form the basis of an interventional design to directly assess the impact of the model on clinical decision-making and hard outcomes such as mortality and LOS.

**TABLE 1:** Characteristics of SafeICU-cohort observational window captured (30 min-4 hours) earlier to Hypothermia. Unless otherwise stated, all values are mean (SD), *significance level at *p*-value >= 0.01, W represents Wilcoxon rank-sum test (non-parametric), this is utilized once the normalcy assumption has been tested. The Chi-squared test of proportions is denoted by the letter C in the table.

Variable	Hypothermia Mean (sd)	Non-hypothermia Mean (sd)	*p*- value (Significance, *p*-value < 0.01)
Age (months)	50.63 (52.12)	40.67 (51.15)	1.75 × 10–12 (W)
Arterial-Diastolic BP (DBP), mm Hg	86.8 (6.67)	87.2 (6.43)	0.0592 (W)
Heart rate, per min	129.69 (8.55)	129.84 (8.15)	0.9836 (W)
Respiratory rate, per min	31.45 (5.35)	33.07 (5.5)	0.0005*(W)
Oxygen Saturation	93.37 (3.1)	92.71 (3.31)	0.01269 (W)
Temperature	36.36 (0.51)	37.53 (0.8)	0.0001*(W)
Gender (F%)	43.6%	32.2%	0.0018*(C)

### 3.2 Model performance on time-series features

**FIGURE 2:**
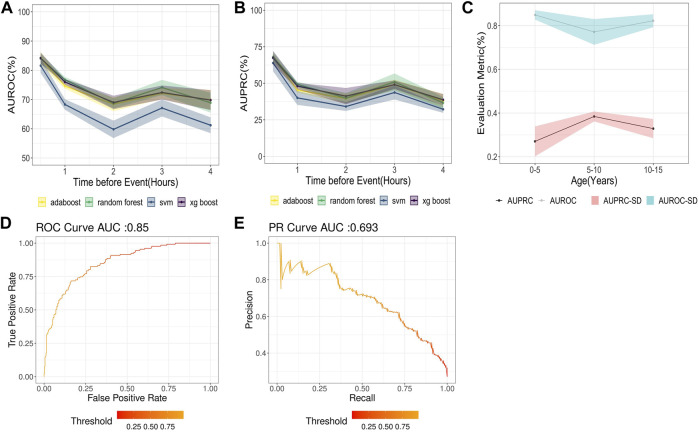
**(A)** AUROC for different models with lead times or times before hypothermia forecast in the next 30 min to 4 h **(B)** AUPRC for different models with lead times or times before hypothermia forecast in the next 30 min to 4 h **(C)** AUROC for the Hypothermia prediction in the next 30 min **(D)** AUPRC for the hypothermia prediction in the next 30 min. **(E)** Results of the AUROC and AUPRC Models for various age groups.

A total of 726 time-series features were found to be statistically noteworthy for variable significance z-scores in the Boruta feature selection analysis for 30 min-4 hours different lead times. Out of the Gradient boosting classifier, Adaboost, Random Forest, and Support vector machine models trained upon these TS-features, the Gradient boosting classifier performed best [Figure 2A,B] with an AUROC of 85% (SD = 1.6) and a precision of 59.2% (SD = 8.8) for a 30-min lead time before the onset of hypothermia (Figure 2D,E). The Average AUROC of 74.28 (SD = 2.26) and AUPRC of 47.56% (SD = 4.4) were achieved for (30 min-4 h) ahead of prediction, with 79.88% (SD = 9.62) of all hypothermia events identified 4 h ahead of their onset. As expected, the model showed a decline in model performance at higher lead times, such as AUROC of 77.2% (SD = 2.3) and precision of 41.34% (SD = 4.8) for 4 h ahead of Hypothermia onset. The findings of all proposed models for predicting hypothermia/non-hypothermia based on physiological factors are presented in Supplementary Table S2. The best model performance indicator (Figure 2C) with an AUROC of 84.2% (SD = 2.1%) is in the age group of “0–5” years. Whereas it slightly decreased in the age group “10–15” years and “5–10” years with an AUROC of 82.3% (SD = 2.9%) and 77.2% (SD = 5.8%) respectively.

### 3.3 Model interpretability analysis

**FIGURE 3:**
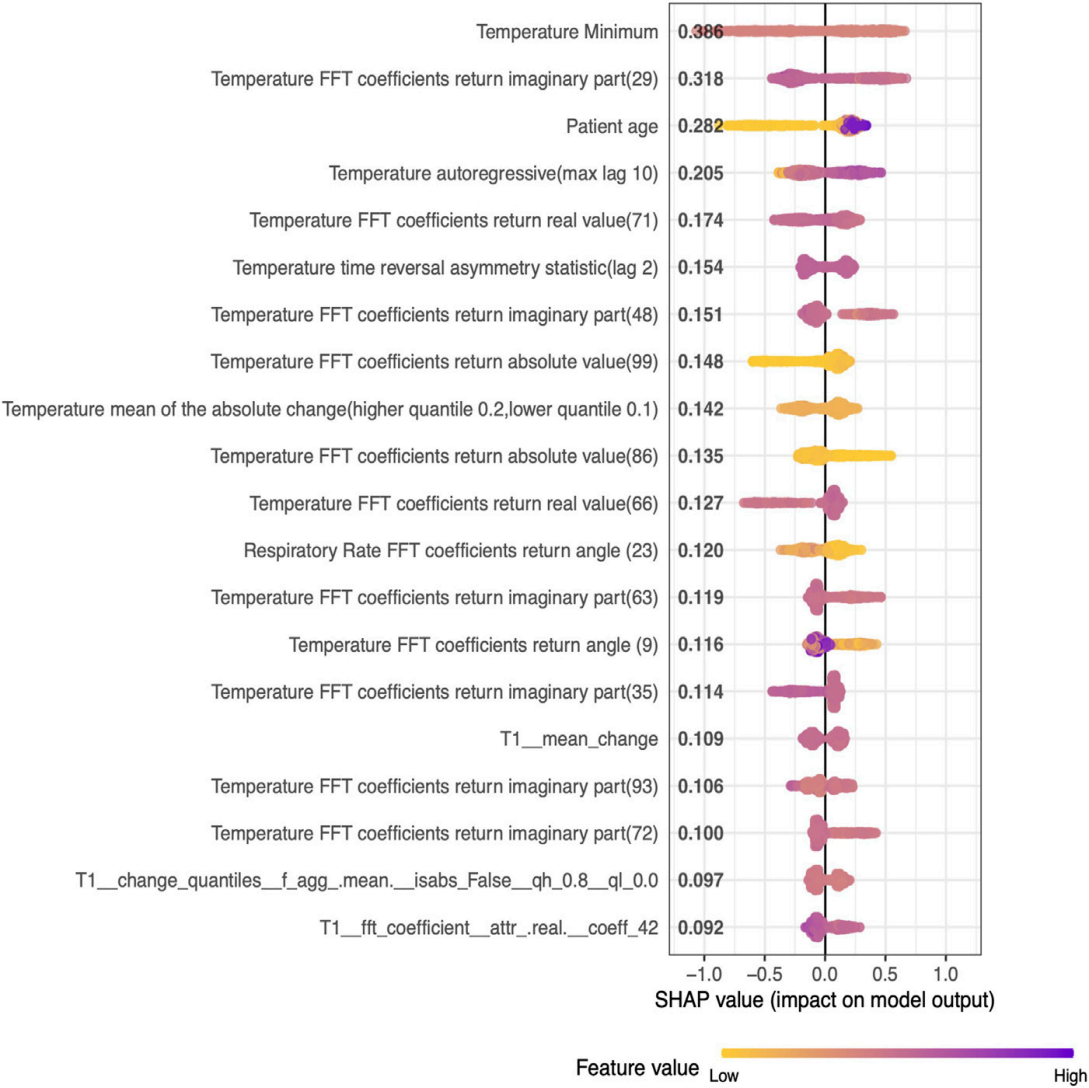
SHAP values of the top 20 Nonlinear features (descending order) generated from the five-fold test set on pediatric data.

The best model captured interpretable and clinically meaningful features such as minimum temperature, age of the patient, mean of absolute change in temperature, and fast Fourier transforms coefficients of respiratory rate (Table 2) as the top predictors for future Hypothermia (Figure 3). Detailed plots of individual top predictors for future hypothermia are available in Supplementary Figure S1. A detailed description of each of the top nonlinear feature’s predictors for future Hypothermia is available in Supplementary Material.

**TABLE 2:** Important non-linear features.

SI. No.	Nonlinear feature	Definition
1	Absolute Energy (abs)	Returns the time series’ absolute energy
2	Continuous Wavelet Transform Coefficients (CWT)	A time scale illustration of a signal is proposed by CWT. The length of the examined signal will aid in dynamically detecting nonlinearities
3	Fast Fourier Transformation Coefficient (FFT)	The Fourier coefficients for the one-dimensional discrete FT are calculated using the Fourier transform algorithm
4	Lag	At lag = 0, a complete correlation will exist for every time series. The correlation value will drop as the time series shifts
5	Mean	The mean of x will be returned by this feature
6	Minimum	The least value among the given collection of values is the minimal number
7	Quantile	The q quantile of x is calculated. Where the quantile divides the sample into equal-sized adjacent subgroups
8	Sum	The sum of the time series values will be calculated
9	Sum of reoccurring data points	This feature will return the total of all time-series data points that appear more than once

### 3.4 Prospective validation of model

**FIGURE 4:**
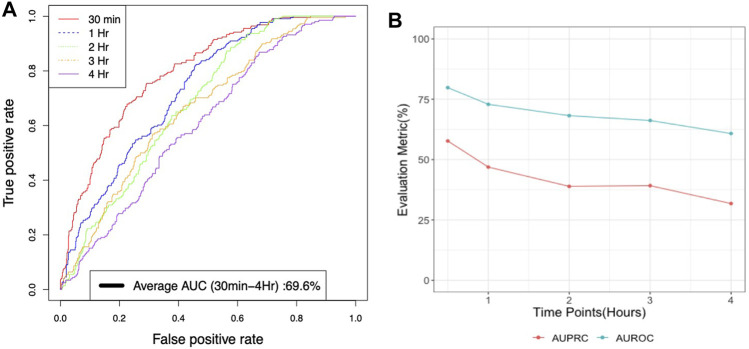
**(A)** Average AUROC performance for xgboost model with lead times or times before for prospective validation of Hypothermia in the next 30 min to 4 h **(B)** AUROC and AUPRC performance for xgboost model with lead times or times for prospective validation of Hypothermia in the next 30 min to 4 h.

Gradient boosting classifier performed best with an AUROC of 79.8% and a precision of 53% for a 30-min lead time before the onset of Hypothermia whereas an AUROC of 69.6% and a precision of 38.52% for a (30-min-4 h) lead time prospective validation of Hypothermia (Figure 4A,B. The findings of prospective validation of a model for predicting hypothermia/non-hypothermia based on physiological factors are presented in Supplementary Table S3.

**FIGURE 5:**
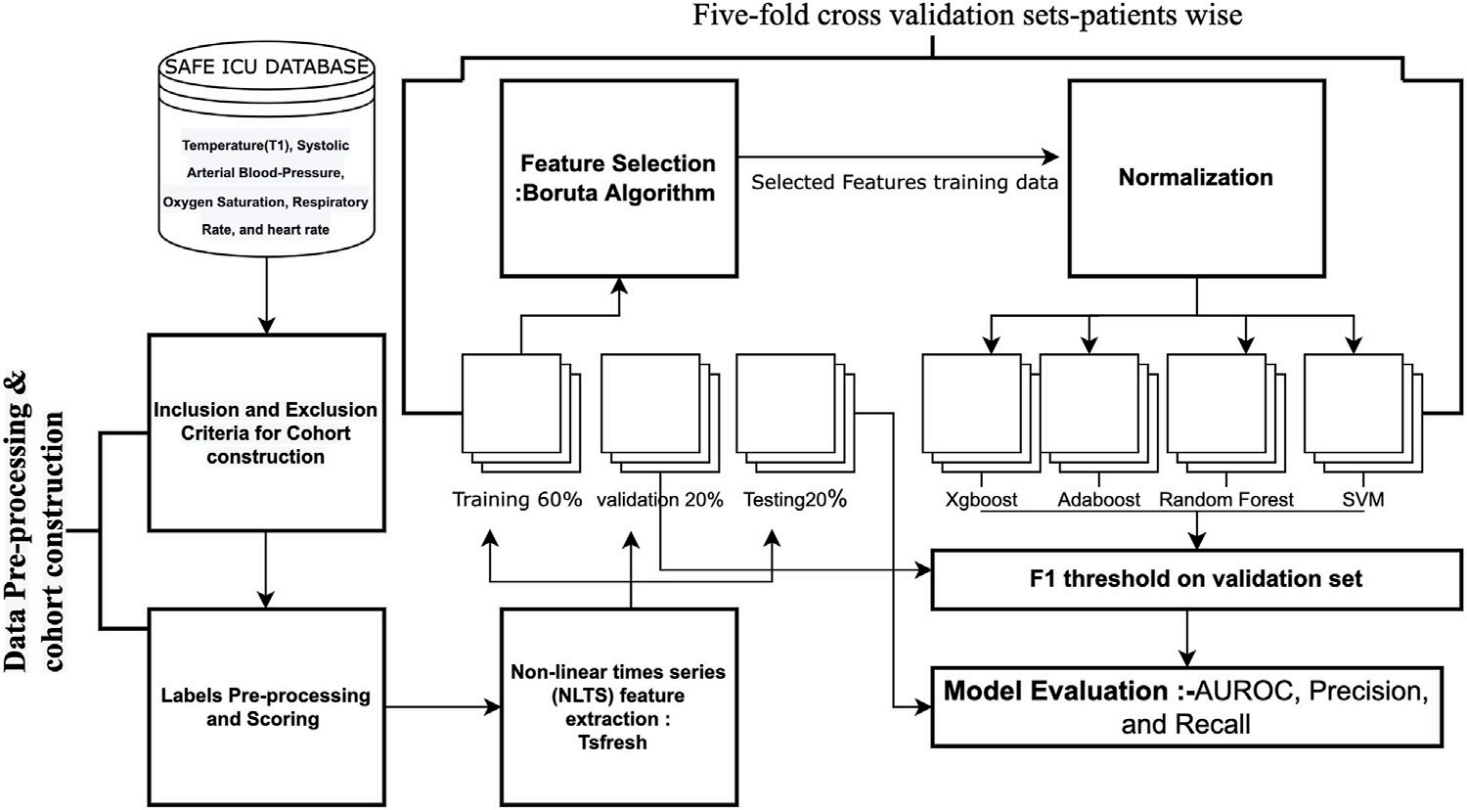
Overview of ThermoGnose Pipeline.

## 4 Discussion

Hypothermia in severe cases can result in mortality; studies have suggested that hypothermic subjects have a significantly higher rate of mortality when compared to non-hypothermia subjects (Kiekkas et al., 2018). Early decision-making can mitigate the risk of hypothermia and associated outcomes, yet, none of the studies has attempted to predict hypothermia. This work presents a first-of-its-kind Hypothermia prediction model in pediatric patients. We leverage a SafeICU (Sethi et al., 2017) resource of pediatric Intensive care unit patients database to build a real-time ML model for the prediction of hypothermia. Our ThermoGnose pipeline predicted the Hypothermia event with an AUC of 85% 30 min ahead of its onset and with an average AUC of 77.6% for 30 min to 4 h ahead of onset. An early therapy decision can help ICU management to initiate therapy such as rewarming to mitigate the risk of hypothermia (Giesbrecht, 2001). The GBM-model performed best at 30 min lead-time and the performances declined during the higher lead times, however, a 30-min window can provide sufficient time for early therapy such as rewarming (Mendrala et al., 2021). Our pipeline produced equal and superior results for the prospective validation, where an AUC of 79.7% was achieved for a lead-time of 30 min to 4 h. Our models were trained on a heterogeneous set of the population having different comorbidities are shown in Supplementary Table S1, enabling our prediction system to generalize to a wide range of Intensive care patients. We also evaluated the interpretability of our models. Since artificial intelligence models suffer from black-box prediction, we kept our approach interpretable using SHAP analysis, which suggests the influence of each predictor on the model output (Lundberg and Lee, 2017). Our analysis suggests that minimum temperature in a 420-min window is the best predictor of future risk of hypothermia (Figure 4).

Since our main aim was to reduce the delay in early identification, we kept our choice predictors on the readily available physiological vitals. These allowed us to make real-time predictions as we have installed in-house software to collect real-time vital data from the bedside at 15 s resolution. Many studies in the intensive care unit suffer from generalizability issues due to the large number of predictors variables required to make predictions and the complexity of the data integration (Wellner et al., 2017). Studies have shown the potential of physiological vital signs for real-time prediction of sepsis. Minimal models have shown good potential for generalizability. We have, therefore, used only five physiological vitals available at the bedside in our models.

Early risk prediction of hypothermia is critical as it can act as a surrogate marker for prognostication of cold diuresis-related hypovolemia (Brown et al., 2012), and early assessment can be helpful for regulating the rewarming cycles (Martin et al., 2015; Vincent et al., 2018) and reducing the length of stay. As we found out that the length of stay (LOS) is associated with the length of stay with hypothermia (Supplementary Figure S2). Therefore, reducing hypothermia by proactive management can help in reducing LOS, Thus, our prediction model can be useful in a wider perspective for clinical practice.

Prediction of hypothermia can reduce the risk of adverse outcomes and mortality in intensive care patients. Early warning systems can help in early treatment and therapy decisions for hypothermia. Our prediction models trained on the cohorts extracted from three million patient hours of data have shown the potential of prospective validation AUC of 79.8% on patients with a wide range of comorbidities; thus, our prediction models have the potential to improve patient care and save lives. The current study and the model, being observational are not geared to evaluate the impact on clinical decision making, which remains the main limitation of our work. However, therapeutic decisions for hypothermia are expected to be less complex, such as the use of warmers and fluids, thus the current model lays the basis for future studies for assessing the clinical impact and outcomes in hypothemic patients based upon ThermoGnose predictions. Importantly, despite continuous monitoring of temperature, the most straightforward vital, predicting hypothermia has remained un-addressed and ThermoGnose provides a starting point for enabling clinical decisions to prevent and proactively treat hypothermia in the pediatric ICUs.

## Data Availability

The raw data supporting the conclusions of this article will be made available by the authors upon reasonable request.
