# Supplementary figures and images for: Early prediction of hypothermia in pediatric intensive care units using machine learning
Source: Front Physiol. 2022 Sep 2;13:921884. doi: 10.3389/fphys.2022.921884 (PMC9511412; doi:10.3389/fphys.2022.921884)

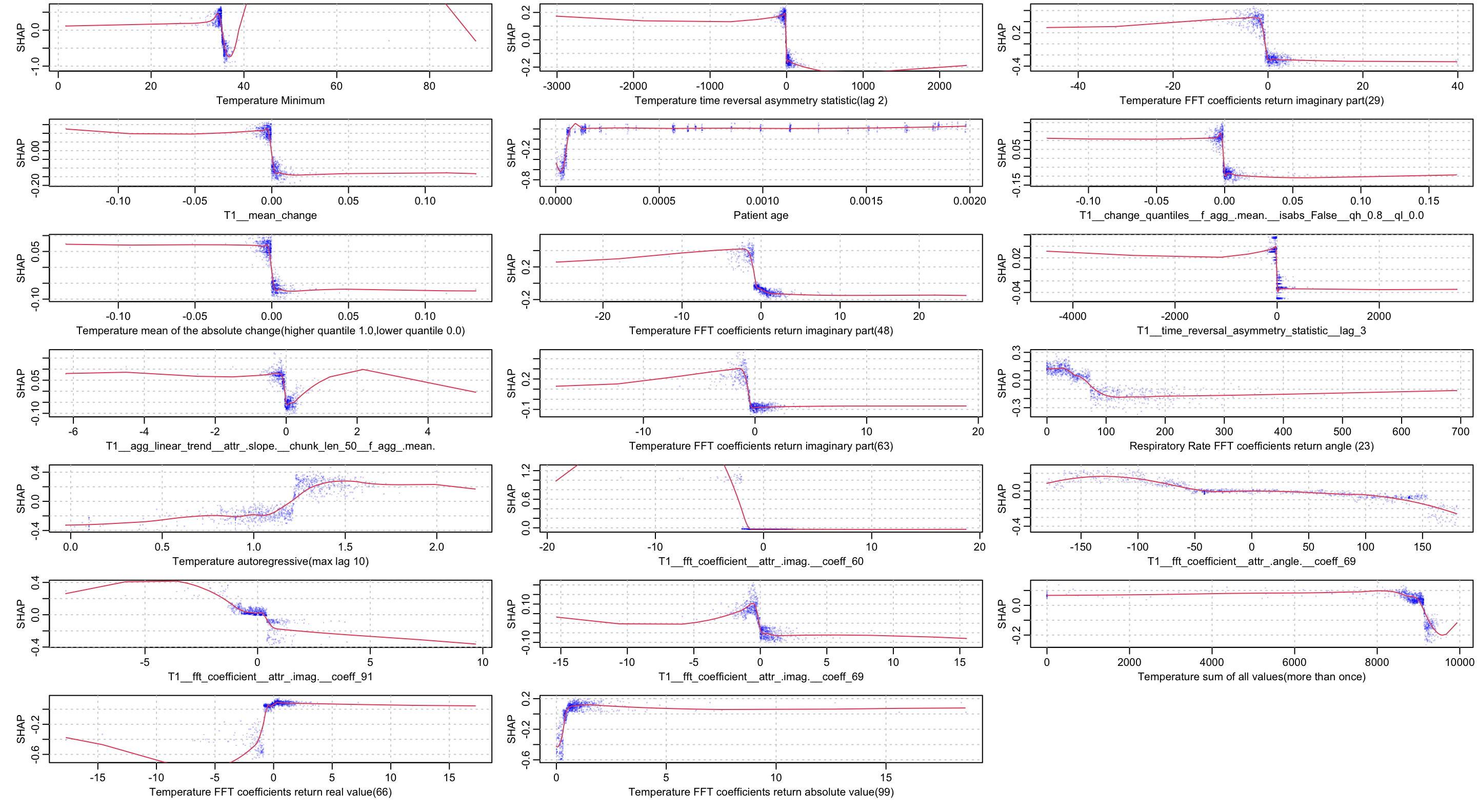

Supplement: Supplementary file 1 [file Image1.TIFF]

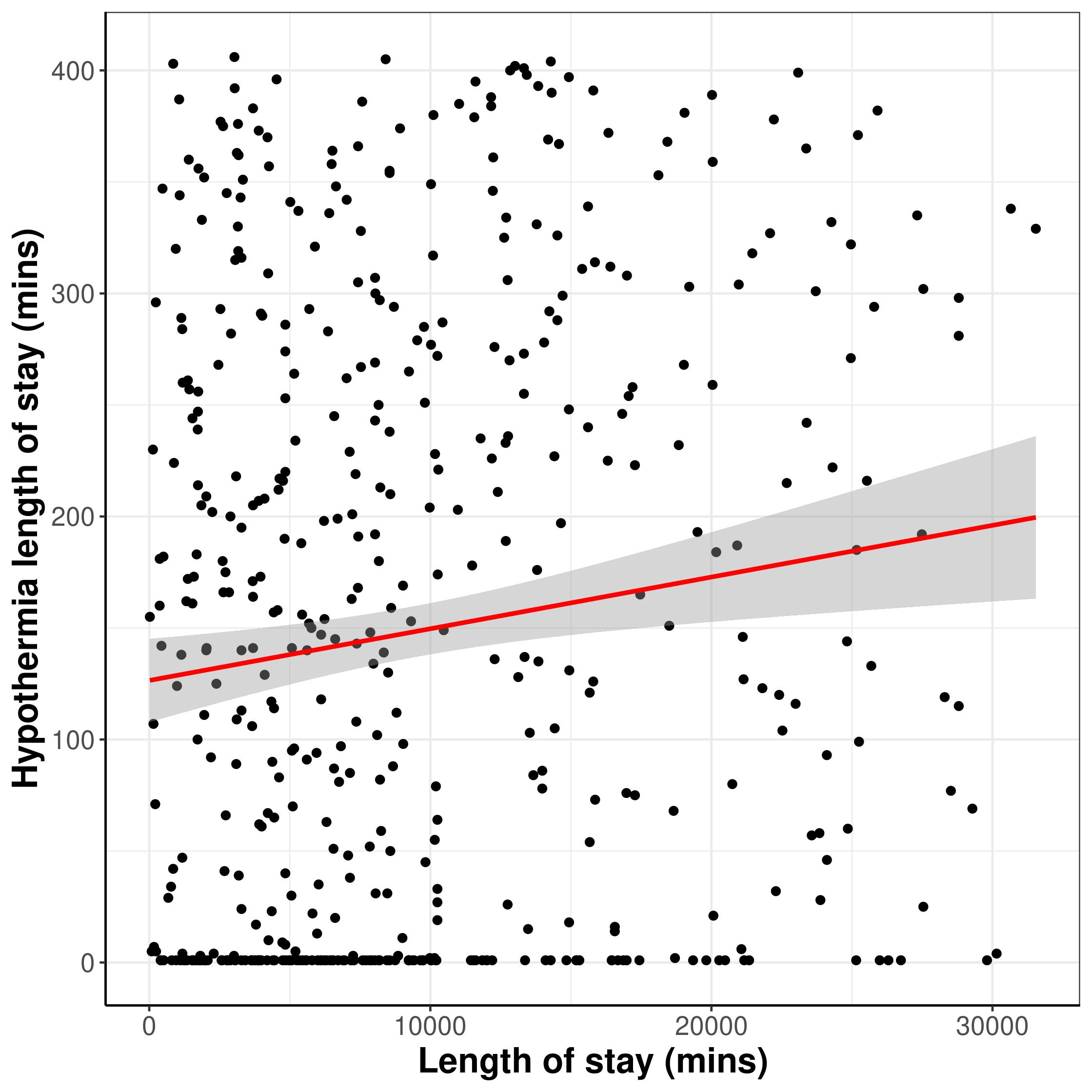

Supplement: Supplementary file 3 [file Image2.JPEG]
