# Supplementary material for: Early prediction of hypothermia in pediatric intensive care units using machine learning
Source: Front Physiol. 2022 Sep 2;13:921884. doi: 10.3389/fphys.2022.921884 (PMC9511412; doi:10.3389/fphys.2022.921884)
Supplement: Supplementary file 2 [file Table1.DOCX]

***Supplementary Material***

# Supplementary Non-linear Feature Description

- **Abs_energy (*x*)**

This option returns the time series' absolute energy, which is determined as the sum over squared values. The feature is calculated using only one argument, x(NumPy.ndarray), which represents the time series. The feature's floating-point value is then returned.

$E=\sum_{i=1}^{n} x_{i}^{2}$

- **cwt_coefficients(*x*, *param*)**

The Continuous Wavelet Transform (cwt) depicts a signal's time scale. The length of the signal under investigation will aid in dynamically detecting nonlinearities. Signal non-linearities are thus exposed in a time bi-frequency domain, which also takes into account non-stationarity. The continuous wavelet transforms for the Ricker Wavelet, or the Mexican Hat Wavelet are calculated using this characteristic. It has two input parameters: x and param. The time series that calculates the feature is x(NumPy.ndarray). And the param (list) comprises the following dictionaries: "widths": x, "coeff": y, and "w": z, according to the dictionaries. Where x is an int array, and y and z are integer types.

This function calculates three parameters such as widths, coeff, and w. This feature collects all of the distinct widths into a single array and then calculates the single cwt for each array chosen. And finally, the values for different coefficients and widths are returned.

This is defined

$\frac{2}{\sqrt{3a\pi}^{\frac{1}{4}}}\left( 1-\frac{x^{2}}{a^{2}} \right)e^{\frac{x^{2}}{{2a}^{2}}}$, where a is considered as the width of the wavelet function.

- **fft_coefficient(*x*, *param*)**

The Fourier coefficients for the one-dimensional discrete Fourier transform are calculated using the Fourier transform algorithm. FT shows all the frequency components and their strength contained in any signal. That is, Fourier analysis is nothing but the extraction of a series of cosines and sines, which, when superimposed, will produce a function. It takes two parameters, x, and the param. Where x(NumPy. ndarray) is the time series used to generate the feature, and param(list) is a list of dictionaries, such as "coeff": x and "attr": s. Here x is an integer type and should be greater than or equal to zero, and the s is a string type and in [“real”, “imag”, “abs”, “angle”].

The resulting coefficients are complex, and this feature can output the imaginary (attr="imag"), real (attr="real"), angle in degrees (attr="angle"), and absolute value (attr="abs").

$A_{k}= \sum_{m=0}^{n-1} a_{m}e^{\left( -2\pi i \frac{mk}{n} \right), k=0,\ldots\ldots n-1}$

- **mean(*x*)**

Mean is considered as a mathematical average of all numbers, and sometimes it is even termed as the arithmetic mean. To calculate the mean, the sum of all the numbers will be divided by the total count of numbers in a set. This feature will return the mean of x. Where x (NumPy.ndarray) is the time series parameter to calculate the feature. It has the floating-point return type.

- **quantile(*x*, *q*)**

The q quantile of x is calculated with this feature. When the quantile divides the sample into equal-sized adjacent subgroups, the sample is divided into equal-sized adjacent subgroups. In this case, the value of x exceeds q percent of the ordered values from x. It requires the input of two parameters, x, and q. Where x (numpy.ndarray) is the feature's time series parameter, and q (float) is the quantile to be calculated. Its return type is floating-point.

- **c3(*x*, *lag*)**

It demonstrates how closely the time series resembles itself. As the values are compared, a perfect correlation will be found for the all-time series at lag=0. The correlation value will drop as the time series shifts. If the time series solely contains random values, the correlation will only exist at lag=0, with no correlation elsewhere.

It requires two inputs: x and lag. Where x (numpy.ndarray) is the time series parameter used to calculate the feature, and lag (int) is the lag utilized in the feature calculation. It returns a value of the floating type.

This function will calculate the value

$\frac{1}{n-2lag}\sum_{i=0}^{n-2lag} x_{i+2lag}^{2}.x_{i+lag}x_{i}$Which will be, $L^{2}(X^{2}).L(X).X]$

- **sum_of_reoccurring_data_points(*x*)**

This feature returns the total of all time-series data points that appear several times. It has only one parameter, x(numpy.ndarray), which is used to compute the feature. It returns the feature's floating-point value.

- **sum_values(*x*)**

The sum of the time series values will be calculated with this function. It has the time series argument x(numpy.ndarray), which is used to calculate the feature. And the value's return type is bool.

- **min_values(x)**

The smallest value in a set of values is called the minimum number. That is, it is either less than or equal to all of the other data values in the collection. The minimum value is a unique value by definition, but in practice, the minimum value might be repeated in the set. The last location of a minimum value of x will be returned by this feature. The length of x will be used to compute this position. To calculate the feature, it uses a single time series parameter x(numpy.ndarray). This feature's floating-point value will be returned.

# Supplementary Figures and Tables

## Supplementary Figures


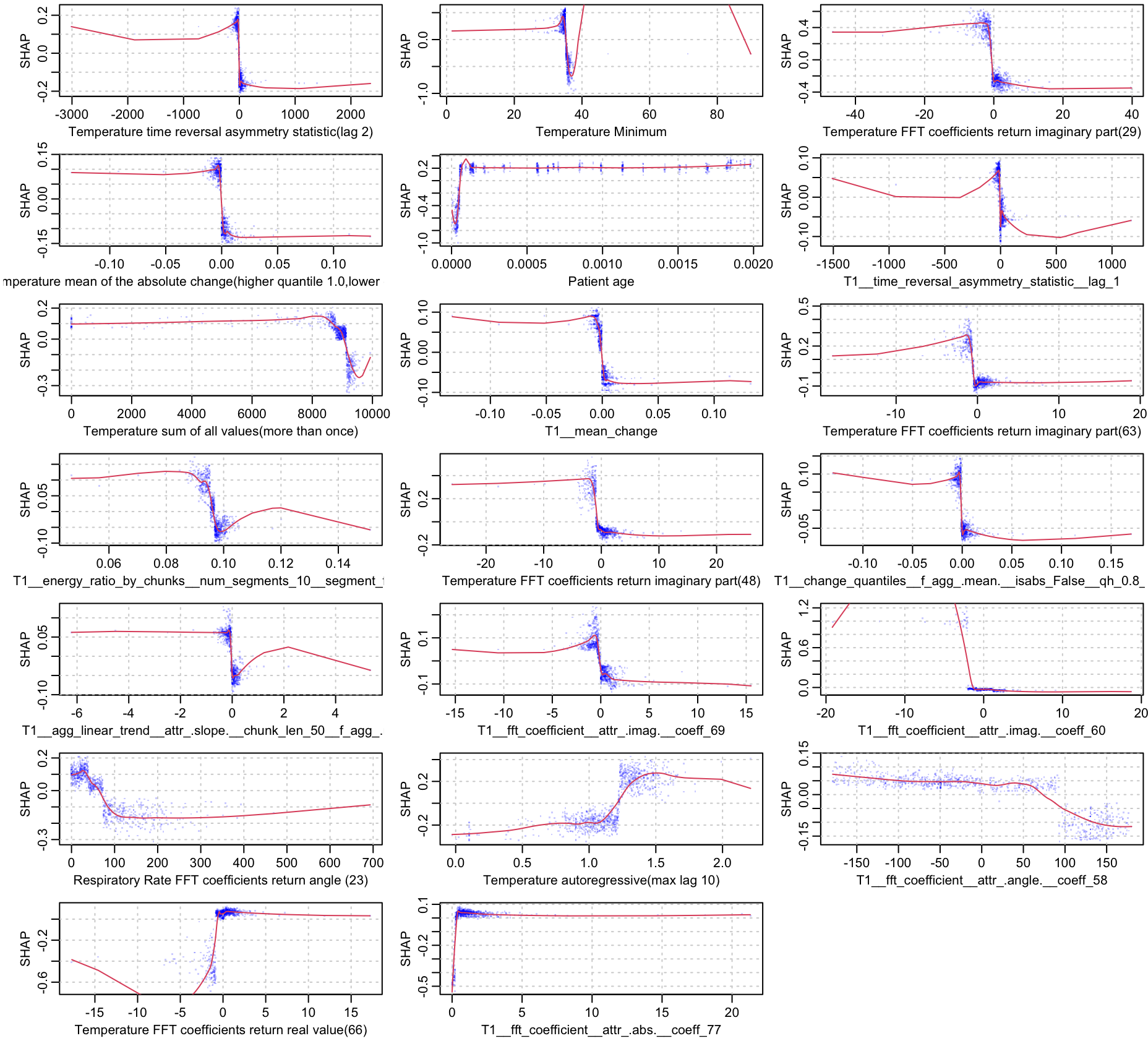


**Supplementary Figure 1.** Detailed plots of individual top predictors for future Hypothermia

##
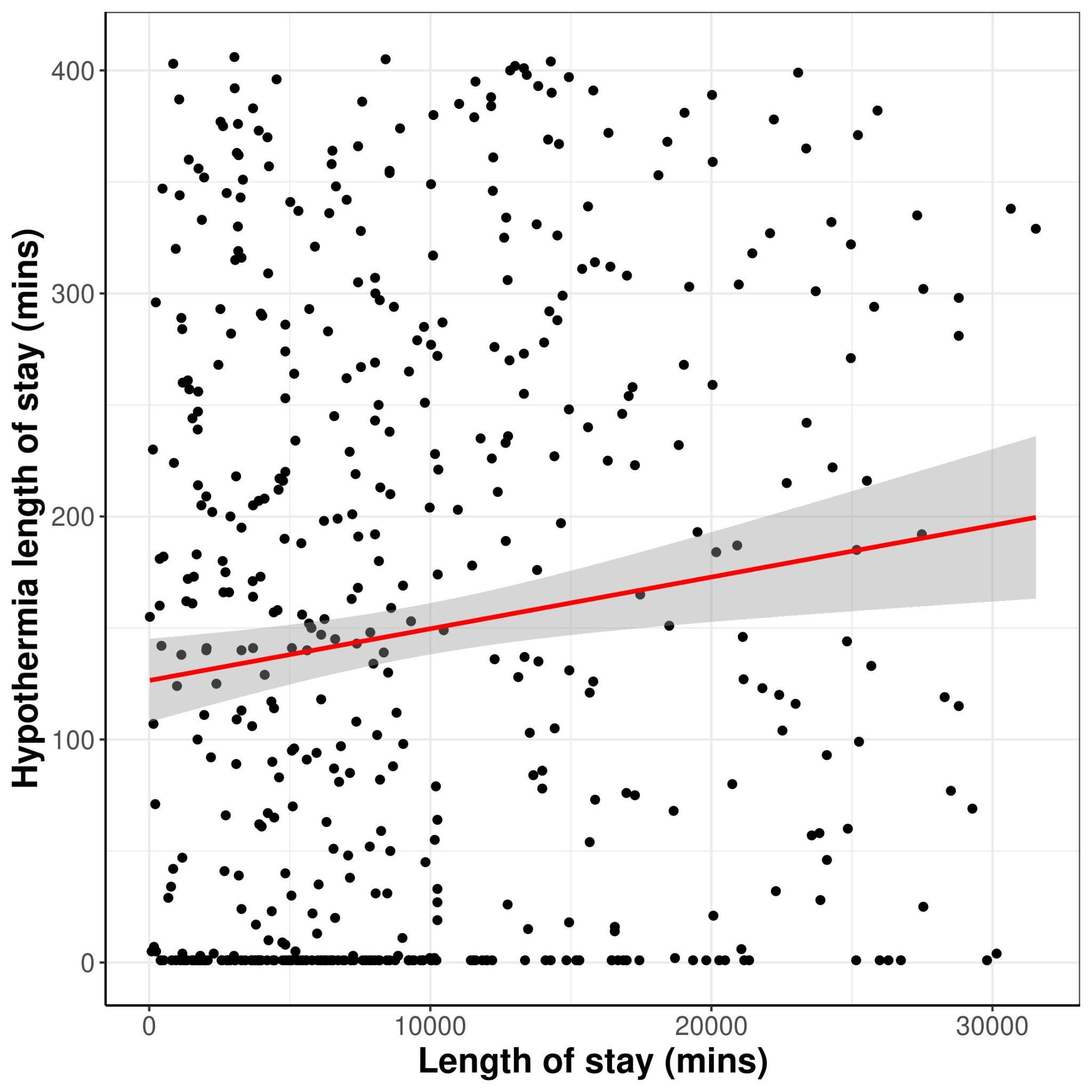


**Supplementary Figure 2.**

## Supplementary Tables

**Supplementary Table 1.** Primary diagnosis in the patients'

| **DIAGNOSIS** | **%** |
| --- | --- |
| **Pneumonia** | **24.24** |
| **Shock** | **23.48** |
| **Sepsis** | **19.7** |
| **Sepsis Shock** | **13.64** |
| **Lower Respiratory Tract Infection** | **12.88** |
| **Encephalopathy** | **12.12** |
| **Congestive Heart Failure** | **10.61** |
| **Ventricular Septal Defects** | **9.09** |
| **Liver Failure** | **6.82** |
| **Tuberculosis** | **6.06** |
| **Respiratory Failure** | **5.3** |
| **Atrial Septal Defect** | **4.55** |
| **Cyanotic Congenital Heart Disease** | **4.55** |
| **Dehydration** | **4.55** |
| **Acyanotic Congenital Heart Disease** | **3.79** |
| **Gastroenteritis** | **3.03** |
| **Diabetic Ketoacidosis** | **3.03** |
| **Systemic Lupus Erythematosus** | **3.03** |
| **Appropriate For Gestational Age** | **3.03** |
| **Preterm** | **2.27** |
| **Patent Ductus Arteriosus** | **2.27** |
| **Multiple Organ Dysfunction Syndrome** | **2.27** |
| **Diarrhea** | **2.27** |
| **Immunodeficiency** | **2.27** |
| **Acute Febrile Illness** | **2.27** |
| **Transposition Of The Great Arteries** | **2.27** |
| **Encephalitis** | **2.27** |
| **Thrombocytopenia** | **2.27** |
| **Hypertension** | **2.27** |
| **Seizures** | **2.27** |
| **Malaria** | **2.27** |
| **Acute Respiratory Distress Syndrome** | **2.27** |
| **Steroid-Resistant Nephrotic Syndrome** | **1.52** |
| **Failure To Thrive** | **1.52** |
| **Pulmonary Hypertension** | **1.52** |
| **Chronic Kidney Disease** | **1.52** |
| **Epilepticus** | **1.52** |
| **Jaundice** | **1.52** |
| **Pneumonectomy** | **1.52** |
| **Bronchiolitis** | **1.52** |
| **Interstitial Lung Disease** | **1.52** |
| **Asthma** | **1.52** |
| **Coagulopathy** | **1.52** |
| **Aventricular Septal Defects** | **0.76** |
| **Neuroblastoma** | **0.76** |
| **Hypogammaglobulinemia** | **0.76** |
| **Laryngotracheomalacia** | **0.76** |
| **Hypoparathyroidism** | **0.76** |
| **Hepatosplenomegaly** | **0.76** |
| **Dengue** | **0.76** |

**Supplementary Table 2.**

Shows the results of the proposed models for predicting hypothermia/non-hypothermia based on physiological variables. The Time Pt. column shows the hours that passed after the observation was taken and the findings were recorded. Models used are XGboost, RF - Random Forest, Adaboost, and SVM - Support Vector Machine. Metrics used for the prediction are as follows: H/NH - Number of Hypothermia/Non-Hypothermia sequences present, AUPRC - Area Under Precision-Recall Curve, PPV - Positive predictive value, AUROC - Area Under Receiver Operating Characteristics, D - Detection, NPV - Negative predictive value, and P - Prediction

| **Time Pt.** | **AUPRC** | **AUROC** | **Accuracy** | **Sensitivity** | **Specificity** | **PPV** | **NPV** | **F1 Threshold** | **Model** |
| --- | --- | --- | --- | --- | --- | --- | --- | --- | --- |
|  | **Mean (SE)** | | | | | | | **Mean** |  |
| 30min(p) | 68.8(6.6) | 85(1.6) | 78.7(4.7) | 70.7(9.7) | 81.5(9.1) | 59.2(8.8) | 88.9(2.6) | 0.6 | XGboost |
| 1hr (P) | 49.3(4.1) | 79.7(1.9) | 66.6(5.6) | 82.5(10.3) | 61.3(10.2) | 41.4(3.9) | 92(2.8) | 0.42 | XGboost |
| 2hr (P) | 38.8(5.9) | 75.6(4) | 58.4(9.8) | 90.2(8.5) | 49.7(14.3) | 34.2(6.9) | 95.3(2.5) | 0.38 | XGboost |
| 3hr (P) | 44(2) | 72.1(1.5) | 65.7(3.3) | 67.1(8.5) | 65.2(7.3) | 40.3(2.7) | 85.3(2.1) | 0.51 | XGboost |
| 4hr (P) | 36.9(3.6) | 73.7(2.3) | 55(4.7) | 88.9(11.1) | 45.4(8.8) | 31.6(1.9) | 94.4(4.5) | 0.33 | XGboost |
| 30min(p) | 66.7(6.4) | 84.5(1.4) | 51.9(2.3) | 46.5(4.7) | 53.8(4.6) | 26.6(3) | 73.7(2.9) | 0.53 | RF |
| 1hr (P) | 51.8(3.6) | 80.6(2) | 69.2(5.2) | 80.6(10.4) | 65.1(9.9) | 43.3(2.9) | 91.8(2.9) | 0.46 | RF |
| 2hr (P) | 38.4(5.6) | 74.8(3.4) | 62.8(6) | 77.9(5.3) | 58.5(8.8) | 34.7(5.2) | 90.6(1.2) | 0.48 | RF |
| 3hr (P) | 45.2(2.1) | 74(1.5) | 64.4(7) | 71.7(12.8) | 61.7(13.7) | 40.3(3.5) | 87(3.5) | 0.49 | RF |
| 4hr (P) | 37.1(2.3) | 72.7(1.9) | 60.3(4.7) | 75.7(14.8) | 55.7(9.7) | 32.6(1.8) | 89.8(3.6) | 0.47 | RF |
| 30min(p) | 67.4(6.7) | 84.3(1.7) | 77.7(2.8) | 74.5(4.2) | 78.7(4.4) | 56(5.4) | 89.6(1.3) | 0.63 | Adaboost |
| 1hr (P) | 48.2(2.3) | 78.8(2.1) | 70.2(1.3) | 75.9(4.1) | 68.3(1.9) | 43.6(2.8) | 89.8(1.5) | 0.64 | Adaboost |
| 2hr (P) | 37.8(6.1) | 74.5(4.1) | 64.9(4.1) | 74(2.6) | 62.3(4.8) | 35.5(5.3) | 89.6(1.8) | 0.67 | Adaboost |
| 3hr (P) | 43.8(1.2) | 71.7(0.8) | 68.9(1.8) | 55.4(4.7) | 73.6(2.5) | 42.2(2.4) | 82.6(1.7) | 0.72 | Adaboost |
| 4hr (P) | 35.5(2.8) | 72.3(1.8) | 64.1(0.8) | 71.5(7) | 62(2.5) | 34.7(1.4) | 88.6(2) | 0.7 | Adaboost |
| 30min(p) | 62.6(6.9) | 81.9(2) | 74.6(4.6) | 73.1(3.6) | 75.2(7) | 52.1(8.3) | 88.6(2.2) | 0.51 | SVM |
| 1hr (P) | 41(4.2) | 69.6(2.8) | 60.9(7.1) | 68.2(13.9) | 57.9(13.8) | 35(3.3) | 85.5(1.9) | 0.48 | SVM |
| 2hr (P) | 31.2(3.3) | 65.9(2.4) | 54.8(2.7) | 74.3(2.2) | 49.4(3.8) | 29(3.2) | 87.3(2) | 0.45 | SVM |
| 3hr (P) | 41.3(1.7) | 68.6(2.1) | 61.6(4.4) | 65.7(9.2) | 60.2(8.3) | 36.7(3.4) | 83.6(2.8) | 0.47 | SVM |
| 4hr (P) | 32.7(2.3) | 66.5(1.9) | 50.4(9.2) | 78.3(12.6) | 42.4(15.4) | 28.3(2.7) | 87.9(1.7) | 0.46 | SVM |

**Supplementary Table 3.** Results of the prospective validation of model for predicting hypothermia/non-hypothermia based on physiological variables

| **Time Pt.** | **AUROC** | **AUPRC** | **Accuracy** | **Sensitivity** | **Specificity** | **PPV** | **NPV** | **Model** |
| --- | --- | --- | --- | --- | --- | --- | --- | --- |
|  |  |  |  | **Mean** |  |  |  |  |
| 30min(p) | 79.8 | 57.7 | 74.6 | 67 | 77.5 | 53 | 86.1 | XGboost |
| 1hr (P) | 72.9 | 46.9 | 58 | 87.8 | 46.3 | 39.1 | 90.6 | XGboost |
| 2hr (P) | 68.2 | 38.9 | 53.5 | 90.5 | 39.1 | 36.7 | 91.3 | XGboost |
| 3hr (P) | 66.2 | 39.2 | 50.2 | 79.4 | 39.1 | 33.1 | 83.3 | XGboost |
| 4hr (P) | 60.8 | 31.8 | 45 | 87.3 | 29.9 | 30.7 | 86.9 | XGboost |
